# Supplementary material for: Experiences of people with dual sensory loss in various areas of life: A qualitative study
Source: PLoS One. 2023 Sep 8;18(9):e0272890. doi: 10.1371/journal.pone.0272890 (PMC10490834; doi:10.1371/journal.pone.0272890)
Supplement: S1 Appendix — (PDF) [file pone.0272890.s001.pdf]

S1 Appendix. Interview guide.

Before constructing this interview guide, we explored literature on dual sensory loss (DSL), and chose four important life areas to focus the interviews on, namely: access to information, mobility, communication and fatigue.

This interview guide was developed in cooperation with healthcare professionals (i.e. computer trainer, mobility trainer, social worker and educationalist) who often encounter clients with DSL and are experts in the areas we were interested in. The interview guide consists of an introduction and open-ended questions about sociodemographic and medical information, and the four areas of interest: access to information, mobility, communication and fatigue. More specifically, the guide consists of questions regarding which challenges or potential favourable factors people experience in these areas, how they cope with these challenges, and how they use their vision and hearing while performing certain activities. The interview was pilot tested under supervision and with a volunteer with DSL.

Introduction

Thank you for your participation in this study. I will first introduce myself and elaborate on the study and the interview itself.

My name is [name] and I am [profession, institution]. In this study, we aim to explore experiences of people with DSL with regard to access to information, mobility, communication, and fatigue, and how they make use of their vision and hearing in these areas. We can then use this information to improve the care provided to people with DSL. To achieve this aim, we will interview people with DSL about how they experience their daily lives. Your experiences and your opinion will be the most important parts of this interview.

Before we start, I would like to discuss some practical matters with you. The interview will last approximately 1 to 1.5 hours. During the interview you can always decline to answer a question. Please also let me know if you do not understand a certain question, if you cannot understand me properly or if you need a break. I also want to emphasize that there are no right or wrong answers. You can also stop the interview at any time, if you wish to do so. Everything we discuss will be treated confidentially. As described in the information letter, I will make an audio recording of this conversation. The recordings will be transcribed, and they will be destroyed at the end of this study.

I will now discuss the interview itself. I will ask you open questions, and please tell me anything that comes to mind. First of all, we are interested in general information about you and your DSL. Then I will ask you questions about your experiences in the areas of access to information, communication, mobility, and fatigue. We will go through these areas one by one. The questions are similar, yet yield valuable unique information. To ensure that we can discuss all topics, I will keep an eye on the time.

Do you have any questions before we start?

General questions

- Can you tell me something about yourself?
    - Name
    - Age

    - Marital status
    - Hobbies
- Can you tell me something about your vision loss/hearing loss?
    - Which medical condition do you have?
    - When did the symptoms start?
    - How did your medical condition develop over time?
    - Values of visual acuity/visual field/hearing loss
- Which aids do you use to help with your vision loss/hearing loss?

Access to information
- [explanation of the definition of access to information (i.e. smartphone, computer and tablet use and watching television)]
- What are your associations with access to information?
- For example, you are going to move and due to COVID-19 restrictions you are not allowed to go to the city hall. Instead, you are requested to arrange your move online. What do you do?
    - How do you use your vision and hearing in this situation?
- For example, you want to use your smartphone to look up at what time the supermarket closes. What do you do?
    - How do you use your vision and hearing in this situation?
- What influence does access to information have on your life in general?
    - What influence do your vision and hearing have?
- Have your experiences regarding access to information changed over time? If yes, how?

Mobility
- What are your associations with mobility?
- For example, you are on foot and you have to cross a busy street. What do you do?
    - How do you use your vision and hearing in this situation?
- For example, you want to visit a friend and you have to use public transportation to get there. What do you do?
    - How do you use your vision and hearing in this situation?
- For example, you are shopping, but you do not know exactly where the shop you want to go to is. What do you do?
    - How do you use your vision and hearing in this situation?
- What influence does mobility have on your life in general?
- Have your experiences regarding mobility changed over time? If yes, how?

Communication
- What are your associations with communication?
- For example, you are at a birthday party and you want to take part in a group conversation. What do you do?
    - How do you use your vision and hearing in this situation?
- For example, you want to make an appointment with your general practitioner by telephone. What do you do?
    - How do you use your vision and hearing in this situation?
- What influence does communication have on your life in general?
- Have your experiences regarding mobility changed over time? If yes, how?

Fatigue

- What are your associations with fatigue?
- How do you usually plan your daily activities?
  - What is the reason you plan your daily activities this way?
  - Have you always planned your activities this way?
- Which activities are exhausting for you?
  - How do you use your vision and hearing in these situations?
- Which activities are energizing for you?
  - How do you use your vision and hearing in this situation?
- What influence does fatigue have on your life in general?
- Have your experiences regarding fatigue changed over time? If yes, how?
