# Supplementary material for: Experiences of people with dual sensory loss in various areas of life: A qualitative study
Source: PLoS One. 2023 Sep 8;18(9):e0272890. doi: 10.1371/journal.pone.0272890 (PMC10490834; doi:10.1371/journal.pone.0272890)
Supplement: Attachment — Submitted filename: PONE-D-22-21133 SAJ.pdf [file pone.0272890.s002.pdf]

PLOS ONE

Experiences of people with dual sensory loss in various areas of life: A qualitative study

--Manuscript Draft--

**Manuscript Number:**	PONE-D-22-21133
**Article Type:**	Research Article
**Full Title:**	Experiences of people with dual sensory loss in various areas of life: A qualitative study
**Short Title:**	Experiences of people with dual sensory loss in various areas of life
**Corresponding Author:**	Esther Veenman, MSc<br>Amsterdam UMC Locatie VUmc<br>Amsterdam, NETHERLANDS
**Keywords:**	Dual sensory loss; access to information; mobility; communication; fatigue; compensation strategies; qualitative reserach
**Abstract:**	People with dual sensory loss (DSL) seem to have limited ability to compensate for their visual impairment with residual hearing, or for their hearing impairment with residual vision, resulting in challenges in various life areas. The aim of this qualitative study was to explore experiences of people with DSL in various life areas, and how they experience sensory compensation. Semi-structured interviews were carried out with twenty adults with DSL (13 females and 7 males, mean age 47 years). Participants varied with regard to causes of DSL and impairment severity. Sensory compensation and experiences in the areas of access to information, mobility, communication and fatigue were discussed. Interviews were audio recorded and transcribed verbatim. Framework analysis was used to summarize and interpret the data. In the area of access to information, results show that, despite various experienced challenges, participants were in general well able to function with the aid of assistive technology, such as voice command functions. Regarding mobility, most participants were capable of finding their way in familiar environments. However, if the setting was unfamiliar, help from others or navigation applications was required. Participants experienced little issues with having conversations in quiet settings, however, crowded settings were considered very difficult. The final results showed that most participants suffered from fatigue. Carefully considering which daily activities are feasible and having a daily routine helped to cope with fatigue. This study revealed the unique experiences of people with DSL in important life areas. The results suggest that, even though they experience many challenges, they are resourceful in finding compensation strategies. It was, however, hard for the participants to indicate how they experience sensory compensation.
**Order of Authors:**	E. Veenman, MSc
	A.A.J. Roelofs, Dr
	M.L. Stolwijk, MSc
	A.M. Bootsma
	R.M.A. van Nispen, prof.dr.
**Additional Information:**	
**Question**	**Response**
**Financial Disclosure**<br><br>Enter a financial disclosure statement that describes the sources of funding for the work included in this submission. Review the submission guidelines for detailed requirements. View published research	This study was funded by the Visio Foundation (https://visiofoundation.org). The funders had no role in study design, data collection and analysis, decision to publish, or preparation of the manuscript.

Powered by Editorial Manager® and ProduXion Manager® from Aries Systems Corporation

articles from *PLOS ONE* for specific examples.<br><br>This statement is required for submission and **will appear in the published article** if the submission is accepted. Please make sure it is accurate.<br><br>**Unfunded studies**<br>Enter: *The author(s) received no specific funding for this work.*<br><br>**Funded studies**<br>Enter a statement with the following details:<br>• Initials of the authors who received each award<br>• Grant numbers awarded to each author<br>• The full name of each funder<br>• URL of each funder website<br>• Did the sponsors or funders play any role in the study design, data collection and analysis, decision to publish, or preparation of the manuscript?<br>• **NO** - Include this sentence at the end of your statement: *The funders had no role in study design, data collection and analysis, decision to publish, or preparation of the manuscript.*<br>• **YES** - Specify the role(s) played.<br><br>* typeset	
**Competing Interests**<br><br>Use the instructions below to enter a competing interest statement for this submission. On behalf of all authors, disclose any competing interests that could be perceived to bias this work—acknowledging all financial support and any other relevant financial or non-financial competing interests.<br><br>This statement is required for submission and **will appear in the published article** if the submission is accepted. Please make sure it is accurate and that any funding sources listed in your Funding Information later in the submission form are also declared in your Financial Disclosure statement.	The authors have declared that no competing interests exist.

View published research articles from *PLOS ONE* for specific examples.<br><br>**NO authors have competing interests**<br><br>Enter: *The authors have declared that no competing interests exist.*<br><br>**Authors with competing interests**<br><br>Enter competing interest details beginning with this statement:<br><br>*I have read the journal's policy and the authors of this manuscript have the following competing interests: [insert competing interests here]*<br><br>* typeset	
**Ethics Statement**<br><br>Enter an ethics statement for this submission. This statement is required if the study involved:<br><br>• Human participants<br>• Human specimens or tissue<br>• Vertebrate animals or cephalopods<br>• Vertebrate embryos or tissues<br>• Field research<br><br>Write "N/A" if the submission does not require an ethics statement.<br><br>General guidance is provided below. Consult the submission guidelines for detailed instructions. **Make sure that all information entered here is included in the Methods section of the manuscript.**	The Medical Ethics Review Board of Amsterdam University Medical Centers - location VU University Medical Center – determined that the present study is not subject to the Medical Research Involving Human Subjects Act (Dutch law). The study was conducted in accordance with the principles of the Declaration of Helsinki. Written informed consent was obtained from all participants.

Powered by Editorial Manager® and ProduXion Manager® from Aries Systems Corporation

**Format for specific study types**<br><br>**Human Subject Research (involving human participants and/or tissue)**<br>• Give the name of the institutional review board or ethics committee that approved the study<br>• Include the approval number and/or a statement indicating approval of this research<br>• Indicate the form of consent obtained (written/oral) or the reason that consent was not obtained (e.g. the data were analyzed anonymously)<br><br>**Animal Research (involving vertebrate animals, embryos or tissues)**<br>• Provide the name of the Institutional Animal Care and Use Committee (IACUC) or other relevant ethics board that reviewed the study protocol, and indicate whether they approved this research or granted a formal waiver of ethical approval<br>• Include an approval number if one was obtained<br>• If the study involved *non-human primates*, add *additional details* about animal welfare and steps taken to ameliorate suffering<br>• If anesthesia, euthanasia, or any kind of animal sacrifice is part of the study, include briefly which substances and/or methods were applied<br><br>**Field Research**<br><br>Include the following details if this study involves the collection of plant, animal, or other materials from a natural setting:<br>• Field permit number<br>• Name of the institution or relevant body that granted permission	
**Data Availability**<br><br>Authors are required to make all data underlying the findings described fully available, without restriction, and from the time of publication. PLOS allows rare exceptions to address legal and ethical concerns. See the PLOS Data Policy and FAQ for detailed information.	No - some restrictions will apply

Powered by Editorial Manager® and ProduXion Manager® from Aries Systems Corporation

A Data Availability Statement describing where the data can be found is required at submission. Your answers to this question constitute the Data Availability Statement and **will be published in the article**, if accepted.<br><br>**Important:** Stating 'data available on request from the author' is not sufficient. If your data are only available upon request, select 'No' for the first question and explain your exceptional situation in the text box.<br><br>Do the authors confirm that all data underlying the findings described in their manuscript are fully available without restriction?	
**Describe where the data may be found in full sentences. If you are copying our sample text, replace any instances of XXX with the appropriate details.**<br><br>• If the data are **held or will be held in a public repository**, include URLs, accession numbers or DOIs. If this information will only be available after acceptance, indicate this by ticking the box below. For example: *All XXX files are available from the XXX database (accession number(s) XXX, XXX.).*<br>• If the data are all contained **within the manuscript and/or Supporting Information files**, enter the following: *All relevant data are within the manuscript and its Supporting Information files.*<br>• If neither of these applies but you are able to provide **details of access elsewhere**, with or without limitations, please do so. For example:<br><br>*Data cannot be shared publicly because of [XXX]. Data are available from the XXX Institutional Data Access / Ethics Committee (contact via XXX) for researchers who meet the criteria for access to confidential data.*<br><br>*The data underlying the results presented in the study are available from (include the name of the third party*	The data are not publicly available<br>due to their containing information that could compromise the privacy of<br>research participants. A summary of the data is available on reasonable<br>request from the corresponding author. Interview guide is included as supplementary material.

and contact information or URL).<br>• This text is appropriate if the data are owned by a third party and authors do not have permission to share the data.<br><br>* typeset	
Additional data availability information:	

Powered by Editorial Manager® and ProduXion Manager® from Aries Systems Corporation

Manuscript

Click here to access/download;Manuscript;Manuscript submission PLOS ONE.docx

Experiences of people with dual sensory loss in various areas of life: A qualitative study

E. Veenman[1,2*], A.A.J. Roelofs[3], M.L. Stolwijk[1,2], A.M. Bootsma[3], R.M.A. van Nispen[1,2]

[1] Amsterdam UMC location Vrije Universiteit Amsterdam, Ophthalmology, Amsterdam, the Netherlands

[2] Amsterdam Public Health, Quality of Care, Amsterdam, the Netherlands

[3] Royal Dutch Visio – Het Loo Erf, Apeldoorn, the Netherlands

* Corresponding author

E-mail: e.veenman1@amsterdamumc.nl (EV)

Author contributions

E. Veenman: Conceptualization, Formal Analysis, Investigation, Methodology, Visualization, Writing – Original Draft Preparation

A.A.J. Roelofs: Conceptualization, Funding Acquisition, Methodology, Supervision, Visualization, Writing – Review & Editing

M.L. Stolwijk: Formal Analysis, Methodology, Writing – Review & Editing

A.M. Bootsma: Conceptualization, Methodology, Project Administration, Writing – Review & Editing

R.M.A. van Nispen: Conceptualization, Funding Acquisition, Supervision, Writing – Review & Editing

Abstract

People with dual sensory loss (DSL) seem to have limited ability to compensate for their visual impairment with residual hearing, or for their hearing impairment with residual vision, resulting in challenges in various life areas. The aim of this qualitative study was to explore experiences of people with DSL in various life areas, and how they experience sensory compensation. Semi-structured interviews were carried out with twenty adults with DSL (13 females and 7 males, mean age 47 years). Participants varied with regard to causes of DSL and impairment severity. Sensory compensation and experiences in the areas of access to information, mobility, communication and fatigue were discussed. Interviews were audio recorded and transcribed verbatim. Framework analysis was used to summarize and interpret the data. In the area of access to information, results show that, despite various experienced challenges, participants were in general well able to function with the aid of assistive technology, such as voice command functions. Regarding mobility, most participants were capable of finding their way in familiar environments. However, if the setting was unfamiliar, help from others or navigation applications was required. Participants experienced little issues with having conversations in quiet settings, however, crowded settings were considered very difficult. The final results showed that most participants suffered from fatigue. Carefully considering which daily activities are feasible and having a daily routine helped to cope with fatigue. This study revealed the unique experiences of people with DSL in important life areas. The results suggest that, even though they experience many challenges, they are resourceful in finding compensation strategies. It was, however, hard for the participants to indicate how they experience sensory compensation.

Introduction

People with dual sensory loss (DSL) have visual and auditory impairments. DSL can be regarded as a spectrum ranging from total vision and/or hearing loss to having some residual vision and/or hearing [1], which means that the severity of the impairments can vary within this population. The prevalence of DSL is estimated to be between 0.2 and 2%, and increases with age[2, 3]. Due to global demographic aging, the number of people with sensory loss is expected to rise [4], making DSL an increasingly relevant topic to address in health research and policy.

Previous research has shown that visually impaired people compensate for their vision loss by using their hearing [5]. Likewise, people with hearing loss compensate with their vision [6]. Neurological studies have revealed that the brain areas associated with vision in sighted individuals are used for auditory processing in visually impaired people [7], and vice versa in people with hearing loss [8]. However, having both visual and auditory impairments makes the compensatory function of both senses less obvious, and seems to result in difficulties that may go beyond the consequences of having a single sensory impairment [9].

Various life areas can be affected in people with DSL. The Nordic Definition of Deafblindness states that: “To varying degrees, deafblindness limits activities and restricts full participation in society. It affects social life, communication, access to information, orientation and the ability to move around freely and safely.”[10]. Previous studies offer support for this statement. Regarding access to information, one study identified the problems that people with DSL experience while using technology [11]; for example, when watching television it is difficult to understand speech whilst simultaneously having background music in the television show. Additionally, a review of DSL literature confirms that problems in the

areas of communication and mobility are often experienced by people with DSL [12]. For example, the authors found that difficulties in identifying facial expressions negatively influenced speech understanding. Moreover, challenges with regard to mobility, such as finding one's way and using public transportation, were identified. Another area that can cause challenges for people with DSL is fatigue. Wahlqvist et al. [13] investigated the mental health of people with Usher syndrome type 1 and found that more than sixty percent of the participants had fatigue-related complaints. Various studies have shown that these areas affect quality of life of other populations as well, such as elderly or people with specific diseases [14-17].

Although some studies give valuable insights into the impact of DSL, the experienced problems of people with DSL are still not fully understood. Previous qualitative research on DSL did focus on one or more of the aforementioned areas [16, 18], but were often aimed at specific groups (e.g. elderly [16] or people with Usher syndrome [18]). A small amount of qualitative studies have focused on compensation strategies of people with DSL [19], however, to our knowledge, no qualitative studies have been conducted in which the role of vision and hearing as compensatory senses in DSL was addressed. The aim of this study was to explore experiences of people with various causes and severities of DSL with regard to access to information, mobility, communication, and fatigue, and how they make use of their vision and hearing in these areas. The aim was partly achieved.

Methods

Study design & participants

This qualitative, explorative study was conducted between January and July 2021 in the Netherlands. The COnsolidated criteria for REporting Qualitative research (COREQ) checklist [20] was used to verify that the study was conducted appropriately.

In-depth interviews were conducted with Dutch-speaking adults with varying severities of both vision and hearing loss. To define vision and hearing loss, the following criteria were used: a best-corrected visual acuity of < 0.3 in the better eye, and/or a visual field of ≤ 30 degrees around the central fixation point [21], and a hearing threshold of >25 dB [22]. In a number of cases, we slightly deviated from these norms. As we were interested in as many different combinations of impairment severity as possible, participants who were close to these norms were included as well. Exclusion criteria were non-congenital brain damage, impaired cognitive functioning, or being dependent on alternative communication (e.g. sign language).

Two channels were used to recruit participants. First, health professionals employed by an inpatient low vision service center in the Netherlands (Royal Dutch Visio) were asked to recruit potential participants. In addition to offering support (e.g. computer training and mobility training) to visually impaired people, Royal Dutch Visio has clients with DSL as well. Second, a call for participation was posted on several social media channels (Facebook, Instagram, Twitter and LinkedIn) of Royal Dutch Visio. If people were interested to participate, they contacted the executive researcher, who provided further information, and sent them an information letter and informed consent form. After receiving the signed

informed consent form, an appointment was made to conduct the interview. In total, 20 interviews were planned after which data saturation would be expected.

Interviews

Semi-structured, in-depth interviews were conducted by the first author. An interview guide was developed in cooperation with healthcare professionals (e.g computer trainer, mobility trainer) who often encounter clients with DSL and are experts in the areas of interest. The interview guide consisted of an introduction and open-ended questions about sociodemographic and medical information, and the four areas of interest mentioned earlier (see Appendix S1). More specifically, the guide consisted of questions regarding which challenges or potential favourable factors people experienced in the four areas, how they coped with these challenges, and how they used their vision and hearing while performing certain activities. Interviews were audio-recorded and transcribed verbatim. With participants' permission, their medical records were requested to collect information about their diagnosis, visual acuity, visual field and hearing threshold; most of the retrieved information was less than three years old.

Data analysis

The data were analysed by means of framework analysis [23] and ATLAS.ti 9 software; the analysis was conducted by two researchers (EV and MS). To get familiar with the data, two interview transcripts were discussed. The main themes were determined, which corresponded to the topics of the interview guide. Next, two interview transcripts were open coded, and by means of discussion subthemes were identified. The main themes and subthemes were combined in a codebook, which was used to code two new transcripts. The process of coding and adapting the code book was repeated two times, after which one researcher (EV) coded

the remaining transcripts with the final codebook. During this process, no additional changes were made to the codebook, suggesting that data saturation was reached [24]. Finally, the coded data was summarized and interpreted by means of comparing the data across participants and identifying patterns in the data.

Ethics

The Medical Ethics Review Board of Amsterdam University Medical Centers - location VU University Medical Center – determined that the present study is not subject to the Medical Research Involving Human Subjects Act (Dutch law). The study was conducted in accordance with the principles of the Declaration of Helsinki.

Results

Participant characteristics

Twenty adults (65% female) with DSL participated in this study. Participant characteristics can be found in Table 1. The mean age of the participants was 47.1 years and ranged from 20 to 88 years. Their DSL was caused by various conditions. Visual acuity, visual field loss, hearing loss and the presence of asymmetrical hearing differed among the participants. In 70% of the participants binaural hearing aids were used. In Fig 1, the participants' visual acuity, visual field loss and hearing loss is presented.

Table 1. Participant characteristics (N = 20)

**Participant characteristics**	**Categories**	**N (%)**	**Mean (SD) [range]**
Age		20 (100%)	47.1 (17.1) [20-88]
Gender	Female	13 (65%)	
	Male	7 (35%)	
Living situation	Living with partner or family	12 (60%)	
	Living alone	8 (40%)	
Diagnosis[a]	Disorders of sclera, cornea, iris, ciliary body and lens	3 (15%)	
	Disorders of choroid and retina [incl. Usher syndrome]	11 (55%) [9 (45%)]	
	Glaucoma	3 (15%)	
	Disorders of optic nerve and visual pathways	3 (15%)	
	Other	3 (15%)	
Visual acuity of the best eye	Normal vision (≥ 0.8)	0 (0%)	
	Near-normal vision (0.3-0.8)	10 (50%)	
	Moderate low vision (0.1-0.3)	4 (20%)	
	Severe low vision (0.05-0.1)	1 (5%)	
	Profound low vision (0.01-0.05)	2 (10%)	
	Total blindness (<0.01)	3 (15%)	
Visual field	Peripheral loss	17 (85%)	
	Central loss (5°-10°)	15 (75%)	
	Central loss (0°-5°)	9 (45%)	
Use of visual aids	Cane	18 (90%)	
	Magnifier	5 (25%)	
	Assistive computer software	5 (25%)	
	Guide dog	4 (20%)	

	Sunglasses/cap/hat	4 (20%)	
	Braille display	2 (10%)	
	Assistive device for television	2 (10%)	
Hearing loss of the best ear	0-25 dB	3 (15%)	
	26-40 dB	4 (20%)	
	41-60 dB	4 (20%)	
	61-80 dB	4 (20%)	
	≥ 81 dB	5 (25%)	
Asymmetrical hearing	Yes	8 (40%)	
	No	9 (45%)	
	Unknown	3 (15%)	
Use of auditory aids	Hearing aid monaural	2 (10%)	
	Hearing aid binaural	14 (70%)	
	Cochlear implant monaural	2 (10%)	
	Cochlear implant binaural	2 (10%)	
	Streamer	4 (20%)	
	Remote microphone	1 (5%)	

[a] Participants could have more than one diagnosis

Fig 1. Participants' visual acuity, peripheral visual field loss and hearing loss. The vertical and horizontal lines indicate the norms for having a visual and auditory impairment, respectively (i.e. visual acuity = 0.3 and hearing loss = 25 dB).

Due to COVID-19 restrictions, 10 participants preferred to be interviewed via an online video call or telephone. The other interviews were conducted at participants' homes. In two cases, a speech-to-text interpreter was present at the participants' request. Participants received a gift voucher after finishing the interview. Interviews lasted between 37 and 97 minutes, with an average of 65 minutes.

Access to information

Regarding access to information, participants discussed their experiences related to smartphone, computer and tablet usage, and watching television.

Smartphone

Most participants owned a smartphone, and used it regularly. In general, they were well able to use the applications and look up information on the internet. Participants with peripheral visual field defects preferred using a smartphone over a computer or tablet: *"With the smartphone, and of course that's because it is small, it makes me see it better. The smaller something is, the better we [people with peripheral visual field defects] can see it (female, aged 56)."* Most participants used voice control, a dark mode (i.e. black background and white text), or magnified the font to facilitate smartphone use. However, some difficulties were associated with this as well. For example, some applications did not work properly with magnified text, and the absence of a dark mode in some applications prevented people who are sensitive to light from using the application. Additionally, mistakes were regularly made with the dictation function; spoken words were not always properly transformed into text, and these mistakes could not be detected easily because of the visual impairment. Controlling the smartphone so that a web page is being read aloud came with certain difficulties as well: *"I have to select what I want to have read aloud. So I cannot say: 'read this page aloud'. (...). With an article, I might be selecting the text six times, because I have to select the first piece of text, then there is an advertisement, then I have to select the next piece of text, scroll again, select again. (...) That makes me think: 'well, never mind then' (male, aged 31)."* Another challenge that many participants referred to, was that they had to put in a lot of effort to be able to understand speech when using the screen reader function of their smartphones. However, the possibility of directly connecting their smartphones to their hearing aids or cochlear implants was regarded as useful in this case.

Computer/tablet

Computers and tablets were also regularly used by most participants; for example, to visit internet pages and to send e-mails. Participants with decreased visual acuity preferred using a computer or tablet over a smartphone: *"There are many options on the tablet to magnify things, and you can just tick on it, and it magnifies, so that is really nice. I do almost everything on my tablet nowadays (female, aged 37)."* In contrast, people with peripheral visual field loss often mentioned that they were not able to get an overview over the entire computer/tablet screen, and therefore preferred using a smartphone. Other difficulties associated with computer and tablet use were related to visual aspects, such as being unable to distinguish the icons and constantly losing the cursor on the screen. To cope with this, some participants used supportive software: *"With that program, I can easily change the font size. […] Now, I need a font size of 28 or 35 to distinguish what I am writing. […] The program also has an excellent screen reader (male, aged 88)."* Other participants used a dark mode or a braille display to make computer/tablet use easier. Also, some participants mentioned that they received a computer training from a low vision service; this was considered useful, because they were taught adequate skills to facilitate their computer and tablet usage.

Television

When watching television, participants were able to enjoy well-structured programs, like the news or certain talk shows, because these are often slowly paced and easy to follow. The faster paced types of television content, however, did cause problems: *"Well, when I am watching a movie, (...) when many things are happening, like in an action movie, I overlook half of it. (...) Or when things are happening in the dark, well, I just cannot see it. And I try to, but I notice that I get the feeling that I should not continue watching. It is so exhausting (female, aged 56)."* These difficulties caused some participants to avoid watching television at all. Similar to computer and tablet use, another frequently mentioned problem was that a

large screen is difficult to oversee for people with peripheral visual field loss. To compensate, they used their smartphone or tablet to watch certain programs on a smaller screen. In turn, other participants were forced to sit close to the television to be able to see what was happening. Auditory impairments caused difficulties as well, especially when many people on television were talking simultaneously or when the spoken language was not the same as the participant's native language. However, directly connecting the television sound to one's hearing aids or cochlear implants was helpful. Another difficulty was not being able to easily read subtitles. Certain assistive technology could solve that problem: *"You can change the background [of the subtitles] and the size. [...] I am happy with it, because if there's someone with a white t-shirt [on television], I cannot read the subtitles (male, aged 52)."* Participants also made use of assistive technology that reads the subtitles aloud. Notably, some participants mentioned that they relied on either vision or hearing while watching television. One participant mentioned: *"At 8 P.M., we watch television for a while. That is, I listen to what is being said, and my wife watches (male, aged 88)."* Another participant experienced watching television differently: *"I often watch a soap series with subtitles with my daughter. [...] If there are no subtitles, I cannot understand anything (female, aged 41)."*

Mobility

In the area of mobility, participants were asked how they experienced travelling on foot and by public transportation. Some findings applied to both travelling modes. Most participants indicated to find it easier to travel in familiar environments as opposed to unfamiliar environments. *"In familiar settings, I can move around by myself pretty well. [...] Familiar ways are fine. And 'being familiar' is key; I have to know a route well to be able to walk it by myself. I can't just go to a new place by myself (female, aged 27)."* Unfamiliar environments tended to influence the participants' confidence: *"So you are more insecure, but that is*

caused by dual sensory loss, because you cannot rely on either of the two senses, you know? And that's just frustrating (female, aged 42)." A consequence of this reduced confidence was that some participants avoided going to unfamiliar places alone. Another way to deal with difficulties regarding mobility, was to seek help from low vision services: *"We go to the shopping mall or a large traffic interchange [...] He teaches me to listen to where everything is coming from. [...] Well, signalling, so using your cane when you cross the street, walking upright, [...] because your cane does the work for you (female, aged 49)."* Another general finding was that participants' vision was negatively influenced by bad light conditions. For example, one participant mentioned: *"When it's light, there's no problem, but when it's very dark, I think it's terrible. I really don't see anything (male, aged 74)."* On the other hand, some participants also experienced problems with bright light. To deal with bad light conditions, some participants used sunglasses, a hat, or street lights to navigate. Others, however, avoided travelling when light conditions were not sufficient.

Walking

Most participants were able to move around on foot, although crossing the street was considered a difficult situation. Participants had difficulties with determining the location or direction of approaching cars or cyclists: *"But you have to be able to hear where the cars are coming from and if there is a road divider [...] and if there is a bicycle path. These are difficult things (female, aged 68)."* To make crossing the street easier, participants used a pedestrian crossing or traffic light, or asked others to walk with them. Participants took their time to make sure a traffic situation was safe. Most participants tended to use their hearing to determine whether there was any traffic approaching; hearing aids and/or a cochlear implant were considered helpful in this situation. When they detected a road user with their hearing, they used their sight to locate that road user more precisely. Other problems in traffic that

were mentioned were that other road users or obstacles on the sidewalk were being overlooked at times and that electric cars and cyclists were difficult to hear. Other traffic was also difficult to hear when there was background noise: *"Some you hear, some you don't. […] When it is very crowded, I cannot hear whether a car is approaching. When people are working in the garden here, and you have to cross the street there, I cannot hear it, it is disrupting (female, aged 41)."* Most participants used a cane or guide dog when walking: *"It makes you feel more secure and it makes you walk faster. I really need it; I cannot cross the street without my cane (female, aged 51)."* A cane or guide dog had other advantages as well: *"Look, people have to see that I cannot see it, because, if I didn't have a cane, they would've thought: 'Why is that woman walking so slowly?' or 'Hurry up!' […] You can see that parents grab their children when they are walking in front of me. […] People let you go first. Or if you're hesitating, people ask 'Can I help you?' (female, aged 51)."*

When finding their way, most participants used navigation applications (e.g. Google Maps) on their smartphone. These applications were considered helpful: *"It makes you more confident. The problem when going to a new place is that you don't recognise the buildings, and now you can just turn it on and then at least you're in the right street (female, aged 42)."* A problem some participants mentioned to experience when finding their way, even when using an application, was to find a specific house. A way participants coped with experienced problems when finding their way was to ask other people for help: *"I haven't been to the city centre in a long time. It has completely changed. I do want to go exploring it there, but I want to go with a volunteer. Look, if I've been to a place once, I know the way (male, aged 52)."*

Public transportation

Most participants prepared their journey before travelling with public transportation. *"I think it is important to make a plan beforehand, to make sure I know what to look out for. […] That you know where you will sit, close to the travel information screen […] and to the exit. The further you go, the more obstacles you can run into (male, aged 27)."* Some participants were able to use their eye sight, which made using public transportation easier. For example, they were able to differentiate buses and could read travel information displays at train stations. Others were not able to use their eye sight for this purpose. As a solution, they used smartphone applications to retrieve travel information and to find their way in train stations. Another way to deal with this was asking others to help them or travel with them, or following other people when stepping out of the train, because this strategy would take them to the station hall. Problems were experienced when trying to understand speech in stations, as this is usually a noisy and reverberating setting. *"I do ask others sometimes, but it's difficult, because often there's a lot of noise and then I can't hear what the answer is (female, aged 52)."* Some participants also had difficulties understanding announcements in the train or bus (e.g. what the following stop was). They had a practical solution for this problem: *"Or when you know beforehand that there will be six stops, and you hear a computer voice six times, then you know [when you have to get out] (male, aged 27)."*

Communication

The area of communication encompassed having conversations face to face and over the telephone. A general challenge that was mentioned, was that other people do not understand the hearing impairment: *"It's the incomprehension of other people, when I say: 'I cannot understand you', they turn up the volume, and still I have to say: 'I still cannot hear you.'. And then they say 'But I am speaking up, right?' Yes, but you're screaming. I find that difficult sometimes (female, aged 56)."* Another difficulty mentioned by some participants

was that it is difficult to see facial expressions or to determine which emotion is expressed by someone's voice: *"Sometimes you make a fool of yourself [...]. Sometimes if someone makes a joke, and then you think [...], that they are being serious and then a misunderstanding occurs (female, aged 52)."* The COVID-19 pandemic also negatively influenced participants' experiences with communication: wearing face masks prevented them from speech reading, and social distancing made it even more difficult to understand another person. Some physical complaints were mentioned as well; as a result of bending towards someone to better understand them, some participants had a bad posture. Communication also affected participants' mental wellbeing: *"There are moments when I can't understand everything and that can be frustrating [...] Sometimes I feel like an outsider. Then I think 'okay, never mind. I will find out later (female, aged 20)."* Others mentioned that they became isolated, because of avoiding social activities.

Face to face conversations

There was one predominant finding regarding communicating face to face, namely that having conversations in a busy environment caused a lot of difficulties. *"For example, in a group where multiple people talk over each other, I cannot handle that. [...] You are expected to smile and enjoy the atmosphere, but I cannot follow the conversation anymore (female, aged 68)."* It caused difficulties as well when there were multiple conversations in the same room at the same time: *"Yes, it is hard for me to focus on these conversations. Especially when the others are talking loudly, and I am talking to someone with a soft voice, I find it difficult to understand that person. [...] So, it is difficult for me when multiple conversations are happening at the same time (male, aged 44)."* The fact that there was often a lot of background noise, made it especially difficult to understand conversations: *"I hear the conversations that others are having, and I try to understand the conversation that I am in,*

but oftentimes there is music playing or something, so it is hard to obtain the information that I want (male, aged 31).” Another difficult situation was when the person talking was too far away to understand what they were saying. Visual aspects caused problems in busy environments as well, for example, it was considered difficult to oversee the conversation and determine who was talking. Light conditions mattered as well: *“If we go somewhere and it is very dim in a living room, this especially bothers me in the winter, if I visit someone who has ambient lighting, that’s a problem for me, because I cannot rely on my hearing, so I have to properly see the person who is talking (female, aged 56).”* The specific difficulties associated with dual sensory loss were mentioned as well: *“I notice that people that I know who have a hearing impairment, they can speech read, and that is not applicable to me, so in a busy environment that’s very difficult for me (female, aged 27).”* Although most participants owned a hearing aid, some participants did not consider them to be very helpful in these situations: *“The hearing aid amplifies everything, so you hear a lot of noise (female, aged 27).”* Others did benefit from their hearing aids, for example because the aid took the direction of their gaze into account. Participants also benefited from other aids: *“If my husband and I go out for dinner, he has a microphone [which is connected to the hearing aid and cochlear implant], then he talks in the microphone [...]. That’s very nice, because I can understand him much better (female, aged 52).”* Other compensation strategies in busy environments were explaining the impairments to others, asking others to clarify or repeat what they said, carrying on the conversation in a quiet place, and withdraw from the conversation. Some participants mentioned that they avoided crowded situations; if they made an exception, they carefully considered their choice: *“That is often the assessment I make when I go to a birthday party: what does it give me and what does it cost me. If there is an equilibrium, it’s not so bad, but if there’s not, you think: ‘I will pass’ or ‘I will go another time’ (male, aged 27).”*

On the other hand, having conversations one-on-one or in a small group (i.e. no more than five persons) in a quiet environment, did not cause many difficulties. *"With four people […], I think: 'wait a minute: is the lighting alright? Yes. Can I understand it? It takes some time getting used to the voices. What is the conversation about?' Well, then it is alright (female, aged 51)."* One important condition to understand the conversation is that people do not speak simultaneously, and that they speak clearly. As opposed to busy environments, most participants did benefit from their hearing aids in quiet situations.

Conversations by telephone

Participants' experiences regarding telephone conversations varied, however, most participants were able to successfully have a conversation by telephone. Still, some difficulties were mentioned: *"Sometimes it's difficult, because sometimes people talk very fast, or sometimes I cannot understand it for some reason (female, aged 52)."* Other challenges that were mentioned were having difficulties remembering a phone number or not being able to understand everything when there is background noise, which can lead to misunderstandings. Again, hearing aids were used to make telephone conversations easier: *"When I call my general practitioner I can hear him directly in my ears [via hearing aids], and I can understand him very well (male, aged 46)."* Other strategies regarding telephone use were turning up the volume, explaining to the other person that they were hard of hearing, or only having telephone conversations in a quiet environment.

Fatigue

Regarding fatigue, participants discussed how they experienced fatigue, what problems it caused, and how they coped with it in their daily lives.

Most participants suffered from fatigue. Some of them experienced fatigue after busy days, others, however, felt fatigued nearly every day: *"It's not something you can skip. It sticks with you. It's not like you get out of bed in the morning and think: 'I'm going to do everything that I want.' You won't make it (male, aged 27)."* Another participant mentioned: *"I try to go to sleep at 10 P.M. […] If I get up at 8 A.M. the next day it's early for me. Often, I still feel tired. […] Some days I think: 'I can never get enough sleep.' But even if my body is well rested, I can still get very tired two hours after I've woken up (female, aged 20)."* Some participants mentioned that their daily energy levels can vary, and that it also depends on the time of day and the time of the year: *"In winter time, […] I don't want to go out in the evening. […] You're already tired in the evening. So when it gets dark at 5 P.M., I have a short amount of time to walk my dog three times, to exercise, to see my friends. […] So in spring and summer, things are going well, […] but when the weather gets worse and it gets darker outside I can do even less (female, aged 42)."* Travelling, communicating in noisy environments, watching television, doing groceries and household tasks were mentioned to be the most tiring activities. These activities were considered exhausting, because they required considerable concentration on vision and/or hearing: *"When we have meetings at work, I am incredibly tired after a two hour meeting. […] When I'm at my desk to continue working, and someone speaks to me, who I should be able to understand, but I can't. […] I am so tired of all the listening that I cannot understand people any more. […] Then I prefer to continue working visually and not talk anymore, because it requires so much effort to understand it (female, aged 52)."*

Fatigue caused several problems in participants' lives. Some participants experienced physical discomforts, like headaches and eye strains. It also influenced participants' mental wellbeing;

fatigue often resulted in frustration, sadness or anger: *"Sometimes I am angry. I think: 'This doesn't make sense at all.' That I just wake up tired, because… Yes, because maybe I've done too much in the previous days. And even though I've gone to sleep early, I'm still tired. […] That's just frustrating sometimes, because you don't want to be tired. You just want to be alert (female, aged 20)."* Participants also tended to compare themselves to others: *"You still want to function like healthy people. Then you quickly tend to do the same as everyone else. […] But not everything is possible (male, aged 41)."*

There were various strategies mentioned to handle fatigue. Some participants received advice from low vision services about balancing their energy, which was considered helpful. Others found it helpful to explain their fatigue to others, so they would encounter less incomprehension. Another strategy was to have a regular sleep rhythm. The most frequently mentioned strategy was making a plan for daily activities, and consider which activities are reachable. *"You have to plan very carefully and consider: what are my priorities and what is important and how am I going to divide that over the day. If I have to do three things in a day, I won't say: 'I will do three things in the morning.' I try to divide it over the day with breaks (male, aged 27)."* Participants often felt most energised in the morning, so they started their day with the most important or tiring activity. Participants also mentioned that it was important to not plan too many activities after a busy day, because they often needed the next day to rest. Rest was considered very important, and most participants usually went to bed early. Most participants also rested in the afternoon: *"Not because I have to sleep, but just not hearing any more, not concentrating anymore, just nothing (female, aged 52)."* Others, however, did need sleep during the day: *"I know that if I go to bed now, I'll be asleep soon. But I think it doesn't matter at what time I do it. Okay, I wouldn't do it in the morning. But in*

the afternoon… Nine out of ten times I go lie down in the afternoon, and I'll be asleep for two hours (female, aged 49).”

Discussion

This study aimed to explore the experiences of people with DSL concerning access to information, mobility, communication, and fatigue in general, and how they utilise their vision and hearing in these areas. The results revealed that participants regularly ran into challenges, but also that they were resourceful in finding ways to cope with them. A notable finding was that participants were mostly unaware of how they make use of their residual vision or hearing to compensate, which made the latter part of the research question hard to answer.

The experiences of people with DSL found in this study do partly overlap with the experiences of people with a single sensory impairment (i.e. a visual or auditory impairment). In the area of access to information, the participants of this study indicated that they often and mostly successfully used multimedia devices; these results are in line with those of a previous study investigating multimedia use of people with a visual impairment. Regarding mobility, previous studies investigating mobility in people with a visual impairment found that they experience challenges in unknown locations [25], a finding that is confirmed by this study. A common problem reported by people with hearing loss is that understanding speech in crowded settings is considered very difficult [26], which is also confirmed by this study. Moreover, some participants mentioned to experience communication breakdown (i.e. being unable to exchange information) in certain situations, a phenomenon also frequently observed in people with hearing loss [27]. Fatigue was previously studied in people with a visual impairment; a cause of fatigue was having to put in a lot of effort to visually interpret the environment [28]. We also found that this was a major cause of fatigue for people with DSL.

In each area we identified challenges that were unique to DSL. For example, in the area of access to information, people with DSL often do not enjoy watching television, a finding supported by a previous study investigating multimedia use of people with DSL [11]. With regard to mobility, our results suggest that people with DSL find it difficult to locate other road users. This was confirmed by another qualitative study investigating independent travel by people with DSL [29]. A finding regarding communication that was unique to DSL, was that they find it difficult to determine the emotion expressed by the speaker, as they both cannot hear it from their voice and cannot distinguish facial expressions. A literature study confirms that difficulties perceiving non-verbal cues can interfere with communication in people with DSL [12]. In the area of fatigue, we found that, on top of the effort needed to perceive the visual world, people with DSL have to put in additional effort to identify what they hear.

Although the experienced problems are mostly unique to each life area, the coping strategies show some overlap. Seeking social support was a frequently mentioned coping strategy in all areas this study focused on. Previous research has demonstrated a positive association between social support and quality of life in other populations [30, 31]. For people with DSL, social support has been identified as a protective factor for depression [32]. Two other coping strategies identified in several areas were the use of aids (e.g. hearing aid, cane) and seeking help from low vision services. Similar to seeking social support, the use of cochlear implants and hearing aids in people with hearing loss was associated with increased quality of life [33, 34]. Although previous research suggests that many people with DSL do not use a hearing aid or cochlear implant (e.g. due to fear of stigmatization) [12], this and other studies indicate that they might benefit from them [35, 36]. A final coping strategy that was present in multiple life areas was avoidance. Although in general this is not considered an effective coping strategy

[37], we found that avoidance was often a well-considered choice. For example, some participants avoided having a conversation in a crowded environment, because it would cost them too much energy. If this is the case, avoidance may be regarded as a reasonable decision. Even though the participants were resourceful in finding coping strategies, the fact that they were in the possession of such a broad range of strategies accentuates the vulnerability of this group.

A strong point of this study is that the qualitative design allowed for a detailed exploration of participants' experiences. Moreover, the mixed sample of participants with regard to gender, age, and severity of DSL contributes to the broadness of the data. A limitation of this study is that nearly half of the interviews were conducted via a video call. In some cases, the internet connections were not stable; as a consequence, these participants' experiences may not have been fully captured. Still, we were able to retrieve valuable information from the audio recordings and the researcher's field notes. The results might also not be generalizable to the entire DSL population as a consequence of the small sample size. However, the final interviews did not provide much new information, which implies that data saturation may have been reached. Lastly, due to the fact that most participants found it difficult to indicate how they made use of sensory compensation in various situations, we gathered less data than expected on this subject. Perhaps participants were unaware of sensory compensation, because it is an integrated part of life and has therefore become an automatic process. Another research design would possibly be better suited to answer this research question, for example by using quantitative methods, e.g. modeling specific visual and hearing functions in relation to daily activities.

In clinical practice, people with DSL often have to visit separate health care professionals for their vision and hearing problems (e.g. low vision services and audiology services) [3]. Therefore, healthcare professionals' expertise of DSL may be lacking. As this may lead to suboptimal care of people with DSL, it is important that professionals are well-informed about possibilities to improve care for people with DSL. For example, promising interventions have recently been developed to improve communication in people with DSL [38] and to reduce fatigue in people with a visual impairment [39]. It would be advisable to adapt and implement the fatigue intervention and existing computer- and mobility trainings to better meet the needs of people with DSL.

As far as we are aware, this is the first qualitative study to investigate the experiences of people with DSL in four important areas of life. Through thorough exploration of participants' experiences, we have revealed challenges and coping strategies that are unique for people with DSL in the access to information, mobility, communication and fatigue. The results of our study may provide healthcare professionals with important information they can use in daily clinical practice.

Acknowledgements

We are grateful to our study participants for sharing their experiences, and to Ronald Pennings, Carel Hoyng and Stichting Ushersyndroom for letting us use data from their CRUSH study. We also appreciate the health care professionals employed by Royal Dutch Visio who helped us develop the interview guide, recruit participants, and retrieve participants’ medical information.

References

1. Dammeyer J. Deafblindness: A review of the literature. Scand J Public Health. 2014;42(7):554-62.

2. Minhas R, Jaiswal A, Chan S, Trevisan J, Paramasivam A, Spruyt-Rocks R. Prevalence of Individuals with Deafblindness and Age-Related Dual-Sensory Loss. J Vis Impair Blind. 2022;116(1):36-47.

3. Vreeken HL, van Rens GH, Knol DL, van Reijen NA, Kramer SE, Festen JM, et al. Dual sensory loss: A major age-related increase of comorbid hearing loss and hearing aid ownership in visually impaired adults. Geriatr Gerontol Int. 2014;14(3):570-6.

4. Bourne R, Steinmetz JD, Flaxman S, Briant PS, Taylor HR, Resnikoff S, et al. Trends in prevalence of blindness and distance and near vision impairment over 30 years: an analysis for the Global Burden of Disease Study. Lancet Glob Health. 2021;9(2):e130-e43.

5. Collignon O, De Volder AG. Further evidence that congenitally blind participants react faster to auditory and tactile spatial targets. Can J Exp Psychol. 2009;63(4):287-93.

6. Bottari D, Nava E, Ley P, Pavani F. Enhanced reactivity to visual stimuli in deaf individuals. Restor Neurol Neurosci. 2010;28(2):167-79.

7. Amedi A, Floel A, Knecht S, Zohary E, Cohen LG. Transcranial magnetic stimulation of the occipital pole interferes with verbal processing in blind subjects. Nat Neurosci. 2004;7(11):1266-70.

8. Sadato N, Yamada H, Okada T, Yoshida M, Hasegawa T, Matsuki K-I, et al. Age-dependent plasticity in the superior temporal sulcus in deaf humans: a functional MRI study. BMC Neurosci. 2004;5(1):1-6.

9. Saunders GH, Echt KV. An overview of dual sensory impairment in older adults: perspectives for rehabilitation. Trends Amplif. 2007;11(4):243-58.

10. Nordens Välfärdscenter. Nordic Definition of Deafblindness. [Internet]. Available from: https://nordicwelfare.org/wp-content/uploads/2018/03/nordic-definition-of-deafblindness.pdf. [Accessed 16th June 2022].

11. Wu Y, Lindsay S, Cable J, Jones R, Evans L, Xie X. Digital Media Usage of Sensory Impaired Users in Wales 2018. [Internet]. Swansea: Swansea University; 2018 [updated 11th October 2018; cited 21st July 2022]. Available from: https://cronfa.swan.ac.uk/Record/cronfa45949

12. Jaiswal A, Aldersey H, Wittich W, Mirza M, Finlayson M. Participation experiences of people with deafblindness or dual sensory loss: A scoping review of global deafblind literature. PLoS One. 2018;13(9):e0203772.

13. Wahlqvist M, Möller K, Möller C, Danermark B. Physical and psychological health, social trust, and financial situation for persons with Usher syndrome type 1. Br J Vis Impair. 2016;34(1):15-25.

14. Musselwhite C, Haddad H. Mobility, accessibility and quality of later life. Qual Ageing Older Adults. 2010;11(1):25-37.

15. Nam S-J. Mediating effect of social support on the relationship between older adults' use of social media and their quality-of-life. Curr Psychol. 2021;40(9):4590-8.

16. Heine C, Browning CJ. The communication and psychosocial perceptions of older adults with sensory loss: a qualitative study. Ageing Soc. 2004;24(1):113-30.

17. Jung JY, Lee JM, Kim MS, Shim YM, Zo JI, Yun YH. Comparison of fatigue, depression, and anxiety as factors affecting posttreatment health- related quality of life in lung cancer survivors. Psychooncology. 2018;27(2):465-70.

18. de Andrade Figueiredo MZ, Chiari BM, de Goulart BNG, editors. Communication in deafblind adults with Usher syndrome: retrospective observational study. Codas; 2013;25(4):319-24.

19. Ehn M, Anderzén-Carlsson A, Möller C, Wahlqvist M. Life strategies of people with deafblindness due to Usher syndrome type 2a – a qualitative study. Int J Qual Stud Health Well-being. 2019;14(1):1656790.

20. Tong A, Sainsbury P, Craig J. Consolidated criteria for reporting qualitative research (COREQ): a 32-item checklist for interviews and focus groups. Int J Qual Health Care. 2007;19(6):349-57.

21. World Health Organization. International classification of diseases 11th revision. [Internet]. Available from: https://icd.who.int/browse10/2019/en. [Accessed 21st July 2022].

22. Olusanya BO, Davis AC, Hoffman HJ. Hearing loss grades and the International classification of functioning, disability and health. Bull World Health Organ. 2019;97(10):725-8.

23. Parkinson S, Eatough V, Holmes J, Stapley E, Midgley N. Framework analysis: a worked example of a study exploring young people's experiences of depression. Qual Res Psychol. 2016;13(2):109-29.

24. Green J, Thorogood N. Qualitative methods for health research: London: SAGE; 2018.

25. Lange R, Kumagai A, Weiss S, Zaffke KB, Day S, Wicker D, et al. Vision-related quality of life in adults with severe peripheral vision loss: a qualitative interview study. J Patient Rep Outcomes. 2021;5(1):1-12.

26. Mamo SK, Mayhew SJ, Nirmalasari O, Oh ES, McNabney MK, Rund J, et al. Age-related hearing loss and communication at a PACE Day Health Center. J Am Med Dir Assoc. 2018;19(5):458-9.

27. Laakso M, Salmenlinna I, Aaltonen T, Koskela I, Ruusuvuori J. Open- class repair initiations in conversations involving middle- aged hearing aid users with mild to moderate loss. Int J Lang Commun Disord. 2019;54(4):620-33.

28. Schakel W, Bode C, Van Der Aa HP, Hulshof CT, Bosmans JE, Van Rens GH, et al. Exploring the patient perspective of fatigue in adults with visual impairment: a qualitative study. BMJ Open. 2017;7(8):e015023.

29. Hersh M. Improving deafblind travelers' experiences: an international survey. J Travel Res. 2016;55(3):380-94.

30. Ratajska A, Glanz BI, Chitnis T, Weiner HL, Healy BC. Social support in multiple sclerosis: Associations with quality of life, depression, and anxiety. J Psychosom Res. 2020;138:110252.

31. De Maria M, Tagliabue S, Ausili D, Vellone E, Matarese M. Perceived social support and health-related quality of life in older adults who have multiple chronic conditions and their caregivers: a dyadic analysis. Soc Sci Med. 2020;262:113193.

32. McDonnall MC. Risk factors for depression among older adults with dual sensory loss. Aging Ment Health. 2009;13(4):569-76.

33. McRackan TR, Bauschard M, Hatch JL, Franko- Tobin E, Droghini HR, Nguyen SA, et al. Meta- analysis of quality- of- life improvement after cochlear implantation and associations with speech recognition abilities. Laryngoscope. 2018;128(4):982-90.

34. Ferguson MA, Kitterick PT, Chong LY, Edmondson- Jones M, Barker F, Hoare DJ. Hearing aids for mild to moderate hearing loss in adults. Cochrane Database Syst Rev. 2017;(9):CD012023.

35. Soper J. Deafblind people's experiences of cochlear implantation. Br J Vi Impair. 2006;24(1):19-29.

36. Damen GW, Pennings RJ, Snik AF, Mylanus EA. Quality of life and cochlear implantation in Usher syndrome type I. Laryngoscope. 2006;116(5):723-8.

37. Wadsworth ME. Development of maladaptive coping: A functional adaptation to chronic, uncontrollable stress. Child Dev Perspect. 2015;9(2):96-100.

38. Vreeken HL, van Nispen RM, Kramer SE, van Rens GH. ‘Dual Sensory Loss Protocol’ for communication and wellbeing of older adults with vision and hearing impairment – A randomized controlled trial. Front Psychol. 2020;11:570339.

39. Veldman MH, van der Aa H, Bode C, Knoop H, Hulshof CT, Koopmanschap M, et al. E-nergEYEze, a vision-specific eHealth intervention based on cognitive behavioral therapy and self-management to reduce fatigue in adults with visual impairment: study protocol for a randomized controlled trial. Trials. 2021;22(1):1-11.

Supporting information

S1 Appendix. Interview guide.

Click here to access/download;Figure;Fig1.tif

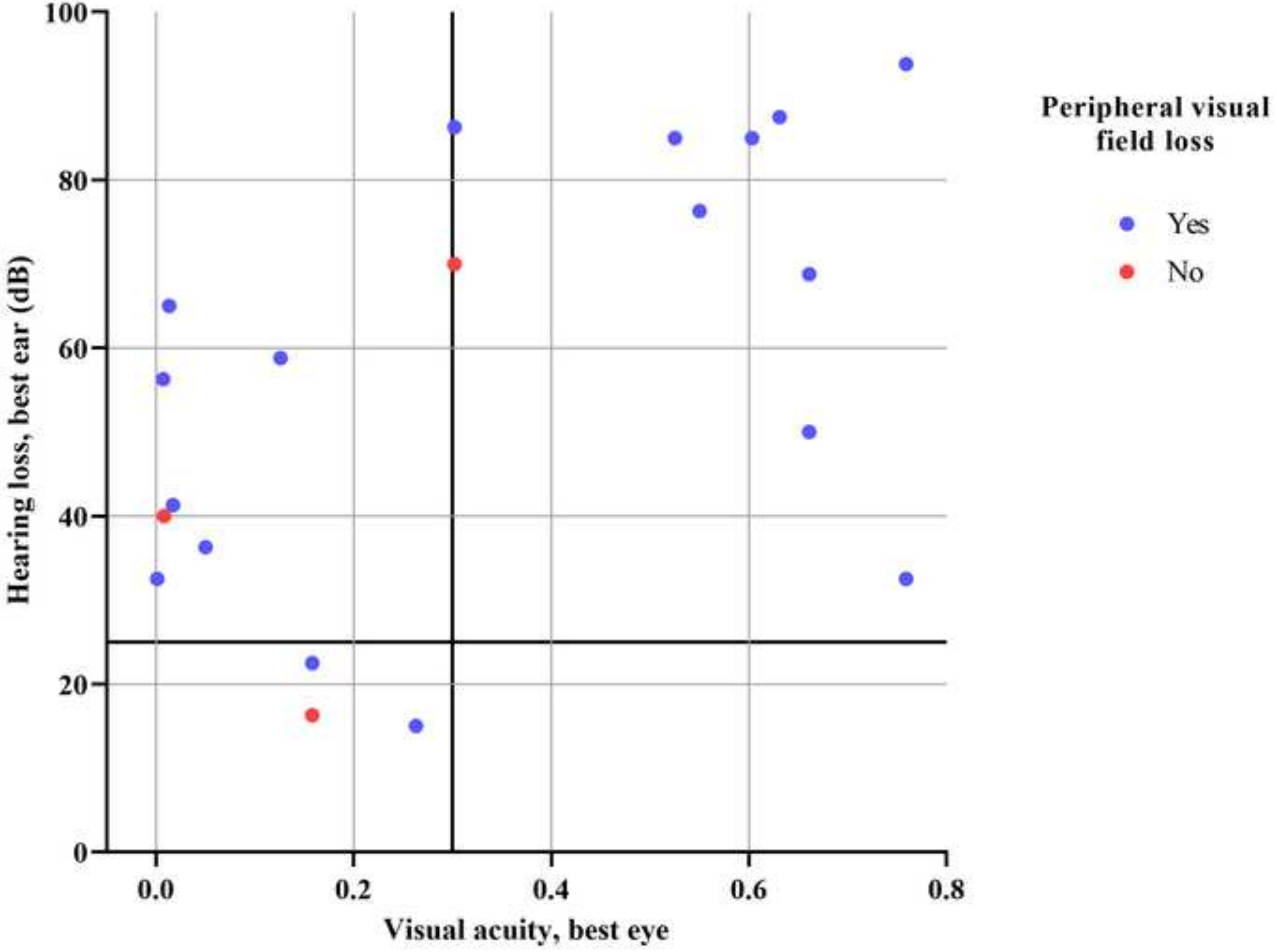

Click here to access/download
Supporting Information
S1_Appendix.pdf
