# Supplementary material for: Experiences of people with dual sensory loss in various areas of life: A qualitative study
Source: PLoS One. 2023 Sep 8;18(9):e0272890. doi: 10.1371/journal.pone.0272890 (PMC10490834; doi:10.1371/journal.pone.0272890)
Supplement: Attachment — Submitted filename: Response to Reviewers_v3.docx [file pone.0272890.s004.docx]

Shazia Khalid, PhD

Academic Editor

*PLOS ONE*

24 August 2023

Manuscript ID: PONE-D-22-21133

Title: Experiences of people with dual sensory loss in various areas of life: A qualitative study

Authors: E. Veenman, A.A.J. Roelofs, M.L. Stolwijk, A.M. Bootsma, R.M.A. van Nispen

Dear Dr Khalid,

Thank you for providing us with the opportunity to submit a second revised version of our manuscript, and for the reviewer’s helpful comments. Below, you can find our point-by-point response to each of the comments. In our revised manuscript, we used track changes to highlight the alterations that were made. We have also included a version of the manuscript without tracked changes. Please note that the page numbers that we refer to in our response refer to the manuscript with tracked changes.

**Comments reviewer 2**

**The manuscript would greatly benefit from a thorough proofreading. There are several instances where grammatical errors, typos, or inconsistencies in punctuation were observed. A careful review of the document is necessary to ensure clarity and readability. Line no. 100, 103, 122, 167, 172, are a few examples for reference**

We asked one of our research fellows who is a native speaker to check the manuscript and we corrected some issues throughout the entire manuscript. A few examples are given below.

*“In-depth interviews were conducted with Dutch-speaking adults, with varying severities of both vision and hearing loss.”* has been changed to *“Comprehensive interviews were undertaken with Dutch-speaking adults, who exhibited diverse degrees of impairment in both vision and hearing.”* (Line 121-122)

*“In total, 20 interviews were planned after which data saturation would be expected.”* has been changed to *“In total, 20 interviews were scheduled which we anticipated to be the number required to achieve data saturation.”* (Line 141-143)

*“In 70% of the participants binaural hearing aids were used. In Fig 1, the participants’ visual acuity, visual field loss and hearing loss is presented.”* has been changed to *“Binaural hearing aids were utilized by 70% of the participants. Fig 1 shows the participants’ visual acuity, visual field and hearing loss information of all 20 participants included in this study.”* (Line 188-191)

**This paper explores the four domains of access to information, mobility, communication, and fatigue. However, the rationale behind the selection of these specific domains is not explicitly stated. It is imperative to thoroughly examine and articulate the significance of addressing these domains in order to enhance the understanding and relevance of the subject matter.**

We agree that the rationale can be stated more clearly. We added the following sentences to the introduction:

*“Deafblind International and both the Nordic and Dutch definitions of deafblindness mention access to information, mobility and communication as life areas that may be problematic for people with DSL [10-12].”* (Line 70-73)

*“The Dutch functional definition of deafblindness identifies fatigue as another important life area that can cause challenges for individuals with DSL.”* (Line 84-85)

**In the introductory chapter, the author presents their findings, which may not be deemed suitable for inclusion in this section. Such findings are typically discussed in the appropriate chapter, such as the discussion chapter. For instance, specific lines, such as 73 and 79, may need to be relocated to the relevant sections where their context and implications can be properly addressed.**

The examples mentioned by the reviewer refer to previous research as opposed to the current study. We adjusted the wording to make this clearer.

*“Additionally, a review of DSL literature confirms that problems in the areas of communication and mobility are often experienced by individuals with DSL [14]. For example, it was found that difficulties in identifying facial expressions negatively influenced speech understanding.”* (Line 80-83)

*“The results of this study showed that more than sixty percent of the participants had fatigue-related complaints.”* (Line 91-92)

**Although the manuscript provides literature support, it is observed that there is a lack of theoretical underpinning in the presented work.**

There has been relatively little research with regard to dual sensory loss. As a consequence, presented theories are limited. 1+1=3 can be regarded as theoretical underpinning for this study, further explained in the paper:

*“However, having both visual and auditory impairments makes the compensatory function of both senses less obvious, and seems to result in difficulties that may go beyond the consequences of having a single sensory impairment; a principle that can be exemplified by 1+1=3 [1, 9].”* (Line 65-68)

In the discussion, we refer back to 1+1=3, in the section highlighting the challenges specific to DSL.

*“In addition to this, we identified challenges that were distinct from challenges reported by individuals with a single sensory impairment. For example, in the area of access to information, individuals with DSL often do not enjoy watching television, a finding supported by a previous study investigating multimedia use of individuals with DSL [13]. With regard to mobility, our results suggest that individuals with DSL find it difficult to locate other road users. This was confirmed by another qualitative study investigating independent travel by individuals with DSL [33]. Related to communication, participants with DSL stated difficulties in determining the emotion expressed by the speaker, due to the fact that both facial expressions and voice could not be distinguished. A literature study confirms that difficulties perceiving non-verbal cues can interfere with communication in individuals with DSL [14]. In the area of fatigue, we found that, on top of the effort needed to perceive the visual world, individuals with DSL have to put in additional effort to identify what they hear. These findings support the theory of 1+1=3, proposing that challenges related to DSL go beyond solely combining those associated with single sensory impairments.”* (Line 523-539)

**In lines 85-86, the author employs the term "aforementioned" instead of using the appropriate and precise wording, which results in an inaccurate narrative. It is important to adhere to the guidelines outlined by the APA style, where authors are expected to use the actual words rather than relying on such general references.**

We agree that this sentence is confusing. We added the life areas that we meant to refer to.

*“Previous qualitative research on DSL did focus on one or more of the aforementioned areas (i.e. access to information, mobility, communication, and fatigue) [19, 21], but were often aimed at specific groups (e.g. older adults [19] or individuals with Usher syndrome [21]).”* (Line 97-100)

**In lines 90-93, the author engages in a discussion regarding the implications of the study, while it is expected that they should focus on articulating the significance of the problem being addressed and emphasizing the importance of providing a viable solution. It is essential to direct attention towards highlighting the relevance and value of both the problem and its corresponding solution within the given context.**

We tried to give more emphasis to the problem in this section.

*“A smaller number of qualitative studies have focused on compensation strategies of individuals with DSL [22], however, to our knowledge, no qualitative studies have been conducted in which the role of vision and hearing as compensatory senses in DSL was addressed. This information might allow individuals with DSL to benefit from strategies (e.g. learn how to use their remaining vision and/or hearing as a compensation strategy) that lead to improved access to information, mobility, communication and to less fatigue. Consequently, this information may also be of importance to healthcare professionals working with the target population as this might help them improve the care they provide.”* (Line 100-108)

**Journal requirements**

**Please review your reference list to ensure that it is complete and correct. If you have cited papers that have been retracted, please include the rationale for doing so in the manuscript text, or remove these references and replace them with relevant current references. Any changes to the reference list should be mentioned in the rebuttal letter that accompanies your revised manuscript. If you need to cite a retracted article, indicate the article’s retracted status in the References list and also include a citation and full reference for the retraction notice.**

We reviewed the reference list and believe it is complete and correct.

We hope we adequately addressed the unresolved issues identified by the reviewer. Thank you for reconsidering our manuscript. We look forward to receiving your response in due course.

Yours sincerely,

On behalf of all authors,

Esther Veenman, MSc

PhD candidate

*Department of Ophthalmology, Amsterdam UMC, location VUmc*

*PO Box 7700*

*1000 SN Amsterdam, the Netherlands*

*Tel: +31-20-4440105*

*Email: e.veenman1@amsterdamumc.nl*
